# How do families access holiday activities and food programmes and other support? Learnings from the Borough of Southwark holiday activities and food club’s evaluation

**DOI:** 10.3389/fpubh.2025.1494180

**Published:** 2025-01-29

**Authors:** Lorraine McSweeney, Anita Sewornu, Louis Goffe, Bronia Arnott, Ashley Adamson

**Affiliations:** ^1^Population Health Sciences Institute, Newcastle University, Newcastle upon Tyne, United Kingdom; ^2^Public Health, Children and Adult Services, London, United Kingdom; ^3^Health Determinants Research Collaboration, Gateshead Council, Gateshead, United Kingdom

**Keywords:** Holiday Activities and Food, intervention evaluation, family support, knowledge of holiday support, school holidays poverty, healthy eating

## Abstract

**Introduction:**

The school holidays can be a challenging time for many families especially for those reliant on free school meals. The Holiday Activities and Food (HAF) programme aims to provide disadvantaged families across England with healthy meals and enriching activities for children and young people. The clubs are usually in the form of a network of independent clubs and often depends on local partnerships and connections, such as the voluntary, community, social and enterprise sectors. In 2023 Southwark Council, interested in building on the findings of a national evaluation and providing more context specific insights relating to access to and use of their clubs, approached the Public Health Intervention Responsive Studies Teams scheme to collaborate in a service provision evaluation.

**Methods:**

As part of the wider evaluation, a qualitative study was conducted. Semi-structured interviews were conducted with parents/carers of HAF eligible children and semi-structured interviews/focus groups were held with children and young people (CYP) attending a HAF club. Recruitment was through the clubs. The transcripts were coded by two independent researchers and thematic analysis applied.

**Results:**

Nine parent/carers and two young people took part in an interview. Six discussion groups with 4–6 young people in each took place. Most participants heard about the club they attended through word of mouth from friends and family, through the child’s school or by seeing a flyer/newsletter advert. Many participants were unaware of the variety and number of different clubs available to families. Finding out about the clubs online was reported to be time-consuming and websites not user-friendly. Parents/carers spoke of the frustration in trying to navigate booking systems. There was also a lack of awareness of the types of other support/signposting available from clubs.

**Discussion:**

The evaluation highlighted the low-level of family awareness of all available HAF activities across the borough. Furthermore, navigating the system was reported as challenging. Southwark Council reviewed and revised its online provision to create a centralised repository accessible to families that aimed to improve awareness and ease the club booking process. Further work is required to improve signposting to support services and provision for parents/carers.

## Introduction

For many families in England, the school holidays are a challenging time, with many facing food insecurity due to loss of Free School Meals (FSM) (1, 2) or increased household expenses during holidays (3). The absence of FSM during holidays can cost families an additional £30–40 a week (1). In 2022/23, the number of people in ‘food insecure’ households rose to 7.2 million, an increase of 2.5 million people since 2021/22, according to data on households that have below average incomes from the Department for Work and Pensions (4). Moreover, 2.3 million people in the UK also lived in a household which had used a food bank in the previous 12 months, a rate of 3%. This includes 6% of children and 3% of working-age adults (5). The Holiday Activities and Food (HAF) programme was first piloted in 2018 by the Department for Education (DfE) to help mitigate such challenges. The HAF aims to provide disadvantaged families across England with healthy meals and enriching activities for their children and young people during the school holidays (2). The scheme provides funding accessible to all local authorities (LAs) in England to provide, or coordinate, such services, usually in the form of a network of independent clubs, which are generally hosted by voluntary sectors or schools, to children and young people aged 5–16 years of age who are eligible for benefits-related FSM over the Easter, summer and winter holidays (6). FSM entitlement is linked to the receipt of particular benefits defined by the English Government (7). Following the COVID-19 pandemic which instigated schools closures, then the cost-of-living crisis which saw a rise in economic hardship for low-income families, the DfE increased the funding for the HAF programme to £220 million per annum until March 2025 (8).

The scheme has a series of objectives for HAF-participating children: eating healthily and being active during the school holidays; participating in engaging and enriching activities that support the development of resilience, character, wellbeing, and education attainment; being safe and not socially isolated; improved health and nutritional literacy; and greater engagement with school and other local services. Furthermore, the scheme aims for families to: develop their nutritional knowledge and food budgeting; and have improved signposting to support services for health, employment and education (6).

In March 2022, the DfE published a national evaluation of the 2021 HAF programme by the independent consultancy Ecorys (2). Ten LAs were randomly selected for the evaluation according to the proportion of children eligible for benefits-related FSM and the number of 5-18-year-olds in each area (2). They found that the programme reached 29% of eligible children who had attended clubs for an average of around 10 days over the summer. These children were more active, confident, and socially active than those who did not attend. They also had engaged in new activities and stated that they had eaten more healthily. Their parent/carers had better access to local services and found it easier to maintain work in comparison to the parent/carers of children that did not attend a HAF club (2). In an evaluation of Northumberland’s County Council HAF 2023, it was found that parents struggled more to find affordable child care in 2023 than in 2022 (9) potentially impacting their ability to work. Low-income families spend a greater proportion of their income on food compared to higher-income families. Whilst low-income families appreciate the importance of a healthy diet, 60% of parents and carers reported they would purchase more fruit and vegetables for their children but were unable to afford to do so (9). The magnitude of income inequalities in child obesity and overweight has been reported to increase between the ages of 5 and 11 years. Lower-income children are more likely to experience upward movements across weight categories than richer ones (10).

Whilst HAF-funded clubs in England, of which LAs have autonomy for coordinating and providing, receive Government funding, the specific delivery/implementation methods can vary based on the resources and needs of each LA (11). Moreover, the success of local clubs often depends on local partnerships and connections, such as voluntary, community, social and enterprise (VCSE) sectors (11). Therefore, there is value in evaluating HAF clubs at a local level to determine barriers and facilitators of successful implementation (8).

In 2023, Southwark Council, interested in building on the findings of the national evaluation (2) and providing more context specific insights of the children and families attending their HAF-funded clubs, submitted their expression of interest for local evaluation of their programme through the National Institute for Health and Social Care Research (NIHR) Public Health Intervention Responsive Studies Teams (PHIRST) scheme (12).

Southwark Council in England, provides grants, funded by the DfE, to HAF club providers to enable them to offer free places to children within the LA who are eligible for benefits-related free school meals. This includes specific funding to support those children with Special Education Needs and Disabilities (SEND). To date, Southwark LA has coordinated with a range of organisations to provide a diverse range of enriching activities to children who attend their HAF. These include schools, youth organisations, tenants’ and residents’ associations, faith groups, football clubs, and children’s centres.

Following an evaluability assessment process (13) Southwark Council and HAF stakeholders identified priority objectives for the evaluation. In response to this, PHIRST Fusion conducted a rapid scoping review (13) of the literature and conducted a mixed methods evaluation. The qualitative evaluation work addressed two of the identified priority objectives: (i) the acceptability and reach of HAF to eligible families and children and (ii), the wider HAF provision/system. Furthermore, whilst these priority objectives were specific for Southwark, the findings of the evaluation will be of interest to other HAF provisions as family-specific views of access/reach of HAF clubs is currently limited.

The aim of this research was: to explore families’ experience of their access to HAF-funded clubs; and capture their awareness of the wider HAF provision across the London Borough of Southwark.

## Materials and methods

### Study design

As part of the wider evaluation, a qualitative study was conducted through focus groups and semi-structured interviews.

### Participants and recruitment

Semi-structured interviews were conducted (by LG and AS) with parents/carers of HAF eligible children and semi-structured interviews/focus groups were held with children and young people (CYP) attending a HAF club.

Parent/carers whose child had attended a club during the summer holidays of 2022 were contacted as part of the wider evaluation and asked to express an interest in taking part in an interview. Those who consented to participate were invited in December 2022 to attend a face-to-face meeting with the lead researcher (LG), and an embedded researcher (AS) from Southwark Council public health team. A second round of recruitment was held in April 2023, when eligible parents/carers who had previously expressed an interest were re-contacted by a staff member from the public health team. New parents/carers were also contacted by scripting an email invitation for HAF providers to send out to those parents/carers whose children attended a club over winter 2022 and Easter 2023.

The initial recruitment for the CYP focus groups centred on the Autumn half term week in October 2022 and was enhanced with a further recruitment period in Spring 2023. HAF clubs were asked to circulate the project information packs and consent forms to parents/carers. Visits were arranged to meet with children, whose parent/carer had consented, at the HAF clubs. A multi-centre approach was taken to recruitment and provided access to two holiday clubs. Interviews/focus groups were led by the lead researcher (LG) and a staff member from the public health team (AS). It was intended that eligible CYP who were not attending a HAF would also be contacted via schools and asked to take part in an interview/focus group, to explore reasons for non-attendance. However, despite best efforts to recruit, this was not possible.

### Data collection

The aim was to interview around 12 parents/carers, or to continue until data saturation was reached. Initially, five parents/carers consented to participate in a face-to-face interview. Following initial data analysis, it was agreed that further data collection was required. Owing to time limitations and to lessen the need for travel and booking venues, the second phase of data collection was completed online by the public health team embedded researcher (AS) using the Teams platform. All participants were asked to provide written and verbal consent (Microsoft forms were used for the virtual interviews).

According to protocol (14), the intention was to deliver 8–12 one-to-one semi-structured interviews with children and young people. Following two initial interviews with young people through one of the HAF clubs during the summer school holidays, the decision was made to adapt the method to a discussion group format delivered by two (one female & one male) researchers; it was anticipated this would ease the flow of conversations. Also included in the sessions was a post-it note exercise to capture desirable activities and as the groups progressed, questions iteratively developed to focus more on how participants discovered the club and their personal and social development.

Interviews and focus groups were audio recorded and transcribed verbatim by a Newcastle University approved transcribing company. Following transcription, recordings were deleted.

### Data analysis

Transcripts were loaded into NVivo 1.6 software (15) and coded by two independent researchers (LM and AS) and the findings discussed at regular project team meetings. A coding frame, which aligned to policy and HAF programme objectives, was developed and agreed. Thematic analysis, using the principles of Braun and Clark (16) was applied to the coded data to determine themes.

## Results

### Parents/carers

Nine parent/carers consented to interview and data saturation was reached. All the participants were female and were from a range of ethnic backgrounds. The interviews lasted between 30 and 45 min. The parent/carers that were interviewed consented to be open and transparent about the impact of the HAF programme on their overall health and wellbeing as parents/carers and on their child. Table 1 outlines the coding framework of the data relevant to the focus of this paper.

**Table 1:** Coding framework for parent/carer interview data.

Code	Description
How they heard about club	Who or what information informed parent about club?
Perception of eligibility criteria	Does the parent know what the criteria for a free place is? Do they feel stigmatised?
Reason for selecting club	Why did parent decide to send child to a specific club?
Selected club	Named club
Club location	Is the chosen club near to where they live?
Preferred methods of communication for information	How does a parent want to hear about a club or be communicated with?
Awareness of other clubs	Does the parent know about other clubs in their area?
Previously attended clubs	Clubs a child may have attended before current club
Sharing information about club	How do families hear about clubs in the wider community?
Sharing information with other parents/carers	Includes word of mouth/WhatsApp
Use of club website	Are parents/carers aware of club website?
Concerns over asking for support	Parent might be thought of not being able to cope
External support	Types of support such as universal credit
Awareness and use of Southwark Council website	Do parents/carers access information/support/services from council?
Potential support from a club	What type of support would a parent be willing to receive/seek from club staff?

There was much discussion regarding access to HAF programmes by parents/carers. In the experience of the participants, for those ‘popular clubs’, allocation of places was made by those first to apply and not by the needs of those living in the club’s catchment area. However, all participants were surprised by the number of clubs that were available throughout the borough when the full offering was presented to them on a map of Southwark.

### Children and young people

Two researchers delivered two semi-structured interviews and six discussion groups across three clubs. There was a total of 32 participants, with groups consisting of 4 to 6 participants. Ages ranged from 9 to 16 years, with most being of younger secondary school Key stage 3 age (11–14 years) (17).

There was a mix of both boys and girls from a range of ethnic backgrounds. The groups also included young people with additional needs, including conditions such as anxiety. Group discussions lasted from 30 to 50 min.

Participants were asked to be open and honest. While the flow and ease of conversation varied across the groups, the researchers were successful in creating a relaxed environment that stimulated conversation. This was validated by comments from some participants who at the end of the discussion, stated they had forgotten that they were being audio recorded.

Table 2 presents the coding framework for the CYP’s data.

**Table 2:** Coding framework for children and young people data.

Code	Description
Club access and awareness	This links to opportunities/action points for the future of the HAF, how, why, and where young people access the clubs
Club discovery	How participants found out about the club that they attended
Knowledge of other clubs	What they know about other clubs in their area/neighbourhood
Club’s community contribution	What is the club’s role/contribution to the local community?

The themes from the parent/carer and CYP’s data were merged into two main themes (i) Access and knowledge of club(s) and (ii), Wider HAF support and provision, they are discussed in turn with anonymised quotes to support the statements.

### Access and knowledge of club(s)

The parents/carers interviewed were made aware about their local HAF club through a variety of different channels, including the child’s school, a flyer advertising the club, or by word of mouth. These channels were also specific to the given club:

*“I mean, it was through my daughter’s school. So, the parent committee coordinator, she put us in contact with [name of club]”* (parent: 1870).

Most of the young people reported that they found out about the club they attended by word of mouth through family and friends, many had older siblings who were attending or had previously attended the club:

*“Because I always used to see my brother coming here. When I went there [club] I saw there was loads of people that I could play with and have fun. When I was a bit older I actually said to come here”* (child: 2).

Some CYP’s mothers received recommendations from friends whose own child attended, others had a family member who had ‘walked past the club’:

*“One day my mum was walking by. She found this youth club because she was going to Tesco, then she found this youth club. Then after it had their number on it so when we went back home she called it and asked if I can join and stuff and that’s how I came in”* (child: 8a).

The particular location of the club did not appear to be a priority in choosing to attend the attended HAF but just of convenience of location to home and/or school:

*“I come here because it’s close to my house”* (child: 7).

Given the extensive number of clubs (37 clubs/providers raising the profile of and advertising of 49 programmes) that were open throughout the summer holidays across Southwark, the lack of awareness of the wider HAF offer was noticeable regarding the availability of those clubs open and accessible to them:

*“I do not know any other clubs, only this one”* (child: 2).

Some parents/carers had an awareness of other clubs, but overall, there was a lack of knowledge of the wider HAF programme:

*“I did not realise there was- I looked on Southwark’s website and saw that there were actually free holiday clubs available. Being a working, single parent is not easy, paying for holiday clubs on top of entertainment during the holidays. So, I was over the moon”* (parent: 0100).

*“Really* [when shown a map of available HAF clubs]*, oh wow, so I did not know, honestly, I did not know…”* (parent: 0103).

Parents/carers that were aware of other clubs, reported finding information about the clubs challenging and time-consuming:

*“Because me spending my time going through and contacting all these different ones, if you have [club] only got 15 spaces I’m going to try and opt for one that’s got 50 spaces. Because between work I do not have time to keep doing all this. I think it would be great if there was a system where you could see, these are the days they operate, here’s the times, if there’s cost involved”* (parent: 1869).

Most parents/carers were unlikely to use the Council’s website to search for local information or those that had, did not find the website user-friendly or accessible:

*“Sometimes if it’s not very clear then I find it really hard to access and you know if you have got to click, click, click, by the time I’ve done all of that I’ve just gave up to be honest. I think it would be good if Southwark had a separate website for this* [club information] *so that you can just Google it and it’s just there, it is its own website. Yes, I think that would be good* (parent: 0100).

When asked how they would like to be informed about the clubs, parents/carers were open to several options such as email, text and the use of WhatsApp. However, the use of non-digital methods such as newsletters and flyers were also a valued option.

### Wider HAF support and provision

Parents/carers reported of additional club benefits out-with the HAF; selected clubs provided support such as homework clubs, reading clubs and help with fixing bikes:

*“My child is in the reading club on Mondays…now we are going to start in January, he starts the reading club”* (parent: 0103).

Some young people also spoke about ‘Take and Make’ boxes whereby they were provided with a box of ingredients with a recipe card to take home and cook with their family (18):

*“…and the good thing is like they give away [Take and Make boxes] to others. I think it’s the charity do things. I’m not sure what it’s actually called, but that’s what they do mainly”* (child: 4a).

As well as the structured ingredient food boxes, some parents/carers also spoke of being gifted left-over food:

*“I mean, food is also important because, at some point, I’ve been given lunch to bring home. And that was really good, and also…. But it’s the [name of club] one, they are also super generous. Because they normally give parents food parcels to bring home, or whatever is available, like vegetables* (parent: 1870).

However, food parcels were not ideal for everyone as one mother described, the foods received were not always culturally applicable:

*“Well, in terms of support I will prefer the finance, as you said, because you know there are times, they give us some foods that it’s not what you eat. Because of our country we come from, not everything here that we eat. We have our own…”* (parent: 0001).

When asked about other types of external support services parents/carers used and what they would value from the clubs, most parent/carers were upfront about benefits they received and the struggles they were facing. They did not feel there was a stigma attached around asking for support, but the majority did not know where they would seek support and had not thought about seeking advice from the HAF staff as they felt the clubs were providing enough already:

*“In all fairness, no. I think they [HAF] do more than enough”* (parent: 1867).

Whilst the ‘stigma’ or asking for support was not an issue for some parents/carers, there was some concern that external agencies may believe parents/carers are not meeting their child’s needs:

*“I think with parents as well you see where I live there’s a lot of single mums, mums what do not work and everything else. Because I used to live on the estate across the road, but I think a lot of parents make do and do go without but you see they do not reach out because they are petrified it’s going to… Do you get what I mean? They’re going to be like, “Oh, you are not meeting your kids need… I [was also] kind of was a bit scared like, “Oh my god. Are they going to take my kid?.” You know?”* (parent: 102).

One mother felt she already received enough support from her community and felt club staff were doing enough:

*“I do not think so [asking for additional support] because it’s enough, for me it’s enough, I have more mums from my community, so we feel like that’s too much help for us because we are working, we have more things to do, especially working. So, it’s very useful, I do not think that we need to ask for more”* (parent: 0103).

It was apparent that most parent/carers were not aware of all the types of support available to them and their families and were unable to say where they would go for help/information. One mother believed that because she was working, there was no other support available to her:

*“Even though I’m a single mum and I’m on a low income I think because I work, I do not think there is any support for me and the kids”* (parent: 102).

When asked about signposting services or support that could be obtained from HAF clubs, parents/carers agreed that club staff signposting or direct support around family challenges such as finances or child behaviour would be acceptable, and they would be open to that:

*“I would be very glad, now. I would be very glad about it”* (parent: 1868).

## Discussion

Families who spoke with us were full of praise for the specific clubs that they attended. However, through the evaluation it was highlighted that knowledge of all the available clubs in the borough was poor restricting the reach to HAF-eligible CYP and families. There is a disassociation between the collective coordination of the HAF provision by Southwark Council and the families to which they are targeted. There is low awareness of what clubs and activities are open and available to families. In essence, the HAF programme is a hyper-localised model, where young people are largely reliant on a parent/carer to identify a suitable specific club, and in turn parents/carers are informed through a combination of school or church disclosure, and serendipity. This disassociation places the emphasis on the clubs, who may be ill equipped, to suitably promote their offerings, and likely contribute to the varying level of attendances from club-to-club.

Given the *ad hoc* and inconsistent methods used to communicate club availability across the authority, both by clubs and Southwark Council, this may well mean that families are unaware of—and subsequently missing out on the benefits that the HAF programme brings to the CYP and parents/carers. It has been reported that schools can be pivotal in helping to market such provision (19, 20) however trust within local communities needs to be well established (20). Some community members may require additional strategies for engagement, such as older children/adolescents (21). In this study, accessing HAF non-attendees for study involvement proved challenging highlighting barriers providers have in engaging certain populations/groups. Evaluations of other LA areas have shown that carrying out yearly evaluations of local HAFs helps to identify reach as well as providing areas for improvement in the following year (19). Both LA officers and researchers should seek out how to build trust with the objective to strengthen community engagement for both the purpose of service delivery and research participation.

During the Southwark HAF evaluation process, it was apparent that providing a central repository of information on the council website about the HAF offer would provide parents/carers with a single point of access to club information, registration and booking. Issues with online booking systems such as an eligibility checker which excluded eligible families (22) and subsequent refinements to improve glitches have featured in other HAFs evaluations (20), highlighting the need for constant monitoring and evaluation of any changes. Gateshead Council HAF also provided a unique link to families in receipt of benefits-related FSM so they can access a hidden part of the council website for ease of pre-booking (23). A broad range of communication methods, inclusive of digital means, are required to help improve awareness of Southwark’s HAF offering to ensure that the borough’s ethnically diverse population are appropriately accommodated. The digital information provided and booking system should be mobile phone compatible as it is likely most families will have access to a phone but not necessarily a tablet or computer. However, to accommodate families who may by digitally excluded, face-to-face bookings should also be available. Not only would this provide information of the wider HAF offer available in the borough but would also likely strengthen the programme on a wider level such as clubs obtaining further funding and so on. Southwark Council were keen to action this evaluation recommendation prior to the new HAF term and a central repository was put in place in September 2023. However, it should also be noted that parents/carers expressed a request that other forms of club information dissemination should also be in place such as leaflets from schools and direct communication through emails/texts would still be appreciated. The use of both formal and informal methods of marketing and communication are apparent through evaluations of other LA HAFs (19). Organisations that had well established links with families in their local communities tended to communicate in a more informal way and told individual families about the availability of places at holiday clubs (19). However as highlighted in Southwark’s evaluation, many families heard about clubs through word of mouth from friends or family (19). Whilst better awareness and attendance of the clubs will require increased provision, Southwark Council have confirmed that at the funding rate provided by DfE to date, the programme could increase the number of attendees while maintaining a quality programme.

A reported aim of the HAF scheme nationally is to ensure families are signposted towards other sources of information and support, such as health services or employment and education opportunities (24) with some HAFs having a dedicated webpage for parents to access links on the HAF website, social media accounts and via email (22). At the time of the evaluation, there appeared to be limited or no signposting through the Southwark Borough HAFs. However, many of the HAFs were likely to operate a drop-off/pick-up model with parents/carers having little interaction with staff. There could be opportunity provided by Southwark Council and HAF clubs to disseminate information on wider support to families via the HAFs. This could include, for example, the offer of parent/carer drop-in days to facilitate access to system-wide support and making use of community ambassadors who would link in with HAF clubs to provide information to families. Clubs in other councils have previously conducted lunch-time sessions for parents/carers where they can access support and guidance on a range of topics (25). However, parents/carers in our study, as in other studies, did express some concerns about seeking external help over fears they are branded ‘inadequate’ as parents/carers (26). Therefore, services which offer practical help to parents/carers, that is provided in non-judgmental ways, that is congnisant of the possible stigmatisation, respecting their concerns are most valued (26). Moreover, it has been suggested that holiday clubs can help foster friendships and a sense of belonging in communities among parents/carers. The involvement of community organisations in the delivery of HAFs is an important aspect. Local organisations understand the strengths and needs of their own neighbourhoods and are likely to have the trust of their community (27). Therefore, clubs can serve an important function in building relationships that increase resilience and well-being in parents/carers (28). The perception of being supported can be as important to parents/carers as any actual support received (26). Building such a network of support/signposting would complement the wider support already offered by Southwark Council relating to alleviating the cost-of-living crisis and increasing food security which includes universal provision of free school meals to all children in primary schools.

Whilst the findings are localised to Southwark Council, many issues and barriers faced are similar to those reported in other LA evaluations. We acknowledge there is a tension between evaluation and research and generalisable knowledge and also a tension between the national HAF programme and (hyper) localised delivery. Hopefully the evaluation itself manages to navigate some of these tensions by providing Southwark stakeholders with specific insights relevant to delivery, but the paper provides more generalisable knowledge to address common barriers that HAFs across many areas will face, such as reach and providing a digital offer. This complements the broad-brush, high level Ecorys evaluation by providing more localised insights—that are still generalisable to other areas (so a mid-level approach).

### Strengths and limitations

One of the researchers, was an embedded researcher from the Public Health Department of Southwark, having someone who was local to the borough conducting the interviews may have facilitated the flow of communication with the residents who took part. Whilst we were able to speak with and obtain the views of CYP and parent/carers from a wide range of ethnic backgrounds we were unable to recruit male parent/carers, and despite best efforts, we were also unable to speak to CYP who chose not to attend a HAF club. If we had been able to speak to these under-represented groups, we may have collected some insightful additional views and experiences of the HAFs. Future research should include male parents/carers at the research design stage to address barriers to participation.

## Conclusion

Whilst those families who attended a HAF club in the London Borough of Southwark in 2022 highly praised the club activities and benefits, the evaluation highlighted the low-level of family awareness of all available HAF activities across the borough. Furthermore, navigating the system was reported as challenging. Southwark Council reviewed and revised its online provision to create a centralised repository accessible to families that aimed to improve awareness and ease the club booking process. Further work is required to improve signposting to support services and provision for parents/carers. Improvements here may foster resilience and support community building among the borough families accessing the HAFs.

## Data Availability

The raw data supporting the conclusions of this article will be made available by the authors, without undue reservation.
